# Changes in Serum Growth Factors during Resistance to Atezolizumab Plus Bevacizumab Treatment in Patients with Unresectable Hepatocellular Carcinoma

**DOI:** 10.3390/cancers15030593

**Published:** 2023-01-18

**Authors:** Zijian Yang, Goki Suda, Osamu Maehara, Masatsugu Ohara, Tomoka Yoda, Takashi Sasaki, Risako Kohya, Sonoe Yoshida, Shunichi Hosoda, Yoshimasa Tokuchi, Takashi Kitagataya, Kazuharu Suzuki, Naoki Kawagishi, Masato Nakai, Takuya Sho, Mitsuteru Natsuizaka, Koji Ogawa, Shunsuke Ohnishi, Naoya Sakamoto

**Affiliations:** 1 Department of Gastroenterology and Hepatology, Graduate School of Medicine, Hokkaido University, Sapporo 001-0021, Japan; 2 Laboratory of Molecular and Cellular Medicine, Faculty of Pharmaceutical Sciences, Hokkaido University, Sapporo 001-0021, Japan

**Keywords:** resistance, mechanism, immune checkpoint inhibitor, atezolizumab, bevacizumab, hepatocellular carcinoma, progressive disease, VEGF-D, angiopoietin-2

## Abstract

**Simple Summary:**

The possible mechanisms of resistance to atezolizumab/bevacizumab for unresectable HCC and the subsequent response to these therapies remain underexplored. The sequential changes in serum growth factors, including VEGF-A, VEGF-C, VEGF-D, ANG-2, FGF-19, HGF, and EGF during atezolizumab/bevacizumab for unresectable HCC were evaluated in 46 patients. Of 32 patients with disease control, 28 experienced PD after CR, PR, or SD with atezolizumab/bevacizumab. Growth factor changes between the baseline and best overall response points (BOR) for patients with disease control showed that FGF-19 significantly increased and ANG2 significantly decreased at the BOR. Growth factor changes between the BOR and the PD point in 28 patients who experienced PD after disease control showed that VEGF-D and ANG2 significantly increased at the PD point compared with that at the BOR. Summarily, increased serum VEGF-D and ANG-2 levels might contribute to developing resistance to atezolizumab/bevacizumab for unresectable HCC and might be target molecules in subsequent salvage therapies.

**Abstract:**

The possible mechanisms of resistance to atezolizumab/bevacizumab for unresectable HCC, and the subsequent response to these therapies, remain underexplored. The sequential changes in serum growth factors, including VEGF-A, VEGF-C, VEGF-D, ANG-2, FGF-19, HGF, and EGF during atezolizumab/bevacizumab for unresectable HCC were evaluated in 46 patients. Patients who experienced PD after CR, PR, or SD to atezolizumab/bevacizumab were evaluated. A total of 4, 9, 19, and 14 patients showed CR, PR, SD, and PD, respectively. Of 32 patients with disease control, 28 experienced PD after CR, PR, or SD with atezolizumab/bevacizumab. Baseline growth factor levels were similar between patients with or without disease control and those with or without an objective response. Growth factor changes between the baseline and the best overall response points (BOR) for patients with disease control showed that FGF-19 significantly increased and ANG2 significantly decreased at the BOR. Growth factor changes between the BOR and the PD point in 28 patients who experienced PD after disease control showed that VEGF-D and ANG2 significantly increased at the PD point compared with that at the BOR. Summarily, increased serum VEGF-D and ANG-2 levels might contribute to developing resistance to atezolizumab/bevacizumab for unresectable HCC and might be target molecules in subsequent salvage therapies.

## 1. Introduction

Hepatocellular carcinoma (HCC) is the sixth most common cancer worldwide and the fourth leading cause of cancer-related deaths [1]. In East Asia, the incidence and mortality of hepatocellular carcinoma have been extremely high [2]. Therefore, the further development of highly effective and safe treatments for HCC is required. To date, several therapeutic options, including hepatectomy, radiofrequency ablation (RFA), transarterial chemoembolization (TACE), and systemic therapy, have been established for HCC. With several successful clinical trials recently, multi-tyrosine kinase inhibitors (TKIs) and immune checkpoint inhibitors (ICIs) have been approved for unresectable HCC, nowadays [3].

Currently, following the approval of TKIs including sorafenib [4], lenvatinib [5], regorafenib [6], and cabozantinib [7], for unresectable HCC, the combination therapy of programmed death ligand 1 (PD-L1) antibody, atezolizumab, and vascular endothelial growth factor (VEGF) antibody, bevacizumab, has been approved in a successful phase 3 clinical trial of IMbrave150 [8]. In the IMbrave150 trial, atezolizumab plus bevacizumab first showed superior overall survival (OS) compared with TKI treatment with sorafenib. Thus, atezolizumab plus bevacizumab has become a first-line systemic therapy for unresectable HCC [9] and has shown high efficacy and safety in patients with unresectable HCC in a real-world setting [10,11,12,13]. Although some patients experienced a durable response to atezolizumab plus bevacizumab, most experienced a progression of their disease after an initial response to atezolizumab plus bevacizumab. The underlying mechanisms of HCC regrowth after a response to atezolizumab plus bevacizumab have not been clarified. Recently, we showed that the efficacy of TKI, lenvatinib [14,15,16], and changes in the serum levels of growth factors, might be associated with the resistance to TKI [17,18]. However, whether this theory can be adapted to ICIs combination therapy has not yet been clarified.

Atezolizumab is an ICI that targets PD-L1 to prevent interaction with PD-1 receptors; thus, suppressing the anti-cancer immune response [8]. Bevacizumab is a VEGF-A inhibitor that binds to VEGFR2 and suppresses tumor angiogenesis [19]. In addition, several reports have shown that VEGF inhibitors can enhance the response of various malignant tumors to the ICI [19]. VEGF inhibitors are reported to promote changes in macrophages from an immunosuppressive to an active form and normalize the tumor vasculature, resulting in increased immune cell infiltration into the tumor [19]. In addition, it has been recently reported that VEGF inhibitors can induce chemokines that promote the infiltration of immune cells into tumors [20,21]. They speculated that one of the possible mechanisms is that anti-VEGF inhibitors induce hypoxic conditions that contribute to increased IFN-γ, resulting in an increase in target chemokines. The anti-VEGF inhibitor, bevacizumab, inhibits VEGF-A by mainly binding to VEGFR2. Thus, we also speculated that if other tumor angiogenic factors increase, including angiopoietin-2 (ANG-2), fibroblast growth factor (FGF), hepatocyte growth factor (HGF), epidermal growth factor (EGF), vascular endothelial growth factor (VEGF)-C, and VEGF-D, it might cause resistance to VEGF-A inhibitors and the combination treatment of ICIs and VEGF-A inhibitors.

In this study, we aimed to analyze the sequential changes in growth factors during atezolizumab plus bevacizumab treatment for unresectable HCC, to gain insights into the mechanism of acquired resistance during atezolizumab plus bevacizumab treatment for HCC.

## 2. Materials and Methods

### 2.1. Patients and Study Design

In this retrospective analysis, patients treated with atezolizumab plus bevacizumab for unresectable HCC between October 2020 and October 2022 at Hokkaido University Hospital were screened. Of those, patients classified as Child–Pugh class A/B, those with unresectable HCC with extrahepatic metastasis, refractory to TACE, or TACE unsuitable [22], were included. Patients who consented to participate in the study were included if they provided complete clinical information; preserved serum for analysis at baseline, best overall response point, and progressive disease point; and had a proper evaluation of their treatment response using dynamic computed tomography (CT) or dynamic magnetic resonance imaging (MRI) at baseline and every 6 to 9 weeks.

We excluded patients who were concomitantly administered atezolizumab plus bevacizumab and other anti-HCC agents, received concomitant use of TACE, were not sufficiently assessed for their treatment response, lacked sufficient clinical data, or had insufficient preserved serum samples for growth factors analysis. 

We analyzed the clinical factors, including age, tumor markers, Barcelona Clinic Liver Cancer (BCLC) stage, laboratory data, liver functional reserve, and serum levels of VEGF-A, VEGF-C, VEGF-D, ANG-2, HGF, EGF, and FGF-19, at baseline, as well as during and after atezolizumab plus bevacizumab treatment. Treatment response was evaluated every 6 to 9 weeks using dynamic CT or MRI according to the Response Evaluation Criteria in Solid Tumors [23]. The best overall response was defined as the best response point across the treatment process. The objective response (OR) rate was defined as the response rate in patients with a complete or partial response evaluated by RECIST [23]. Disease control rates were defined as the response rate in patients with a complete response, partial response, or stable disease, as evaluated by RECIST [23]. 

This study was approved by the ethics committee of the Hokkaido University Hospital (approval number: 020-0204) and the protocol conformed to the Declaration of Helsinki. Written informed consent to participate in the clinical study was obtained from all included patients. 

### 2.2. Analysis of Changes in Serum Growth Factors

We evaluated serum VEGF-A, VEGF-C, VEGF-D, ANG-2, HGF, EGF, and FGF-19, levels using commercial enzyme-linked immunosorbent assays, according to the manufacturer’s protocols. (ANG-2, FGF-19, HGF, and EGF: R&D Systems, Minneapolis, MN, USA; VEGF-A: Cloud-Clone Crop, Wuhan, China; VEGF-C, VEGF-D: Thermo Fisher Scientific, Waltham, MA, USA).

We analyzed changes in serum growth factors at baseline, as well as the best overall response and progressive disease (PD) points. 

### 2.3. Treatment Protocol

During atezolizumab plus bevacizumab treatment, patients were administered atezolizumab (1200 mg) plus bevacizumab (15 mg/kg) intravenously once every 3 weeks. When disease progression was observed, or unacceptable adverse events occurred, treatment was discontinued. The administration of atezolizumab and/or bevacizumab was interrupted if the patients developed grade 3 or higher adverse events (AEs) or unacceptable AEs. Atezolizumab and/or bevacizumab were re-administered according to the package inserts for atezolizumab and bevacizumab when the symptoms were resolved.

### 2.4. Statistical Analysis

The chi-square and Fisher’s exact tests were used to analyze categorical variables and the Mann–Whitney U and Wilcoxon signed-rank tests were used to analyze continuous variables. Statistical analyses were performed using GraphPad Prism version 9.4.0 (GraphPad Software, La Jolla, CA, USA). The threshold for statistical significance was set at *p* < 0.05 for all statistical analyses.

## 3. Results

### 3.1. Patient Characteristics

**Figure 1:**
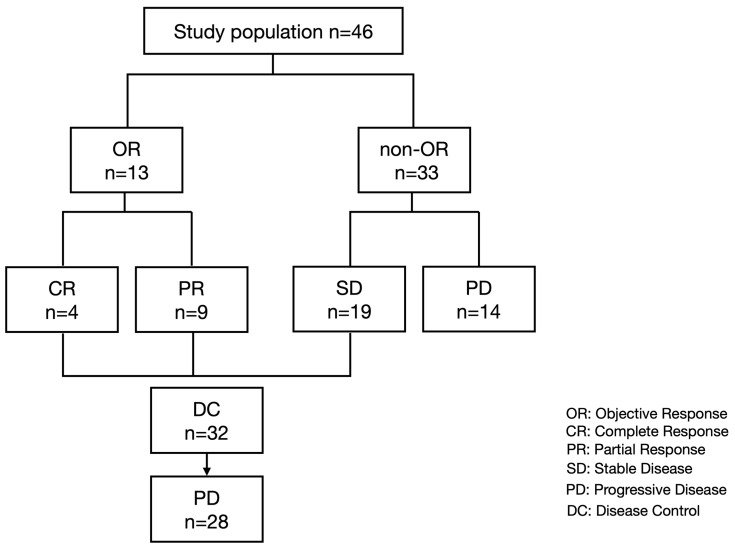
Study flow and treatment response.

We included 46 patients with unresectable HCC who started treatment with atezolizumab plus bevacizumab between October 2020 and October 2022, had proper clinical information, and evaluated the treatment response by CT or MRI. All included patients had preserved serum at the baseline, best overall response point (in patients with stable disease (SD), partial response (PR), and complete response (CR)), and the PD point to evaluate the changes in serum growth factors. As shown in Figure 1, 4, 9, 19, and 14 patients had the best overall response of CR, PR, SD, and PD, respectively. Of the 32 patients with disease control, 28 experienced PD after CR, PR, or SD with atezolizumab plus bevacizumab.

The baseline characteristics of the 46 patients, along with a comparison of the baseline patient characteristics between patients with and without disease control, are summarized in Table 1.

The median age of the recruited patients was 72 years (range: 31–84), and nine (19.6%) were female. The liver disease etiologies of HBV infection, HCV infection, and non-hepatitis viral infection were 15, 6, and 25, respectively. All patients had Child–Pugh grade A disease, and 16 (34.8%) had BCLC stage B disease. In the aspect of tumor markers, the median serum AFP level was 24.05 ng/mL (range: 2.3–57,125.2), and the median serum prothrombin induced by vitamin K absence-II level was 886.5 mAU/mL (range: 19–213,066).

### 3.2. Comparison of Baseline Patients’ Characteristics and Growth Factor Levels between Patients with or without Disease Control or an OR

A comparison of the baseline patient characteristics between patients with and without disease control revealed that the median age was significantly higher in patients without disease control in this cohort.

**Figure 2:**
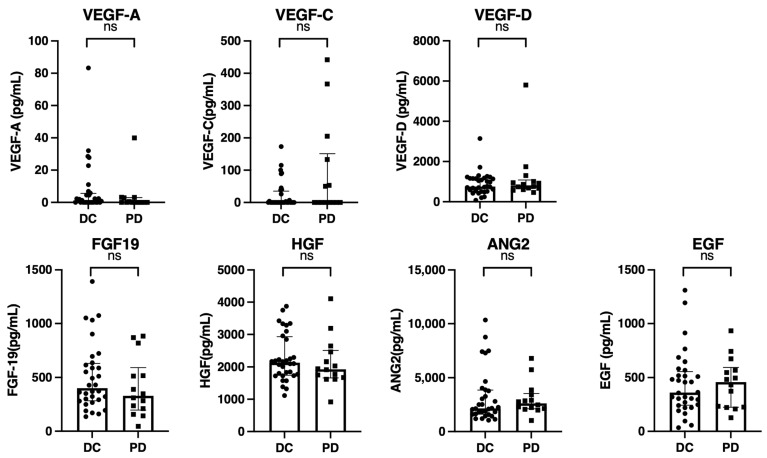
Comparison of baseline growth factors between patients with and without disease control (CR, PR, SD) (*n* = 46). Baseline serum VEGF-A, VEGF-C, VEGF-D, FGF-19, HGF, ANG-2, and EGF levels were compared between patients with or without disease control (CR, PR, and SD). The bar graph shows the median serum growth factor levels with an interquartile range indicating the error bars. ns, not significant; VEGF, vascular endothelial growth factor; FGF-19, fibroblast growth factor-19; HGF, hepatocyte growth factor; ANG-2, angiopoietin-2; EGF, epidermal growth factor; DC, disease control; PD, progressive disease.

We subsequently analyzed baseline growth factor levels and changes in growth factors during atezolizumab plus bevacizumab treatment. Figure 2 shows a comparison of the baseline growth factors between patients with and without disease control. As shown in Figure 2, the baseline growth factor levels were similar between patients with and without disease control.

Subsequently, we compared the baseline patient characteristics and growth factor levels between patients with and without an OR. As shown in Table 2, our findings revealed that the median ages were significantly higher in patients without an OR in this cohort.

**Figure 3:**
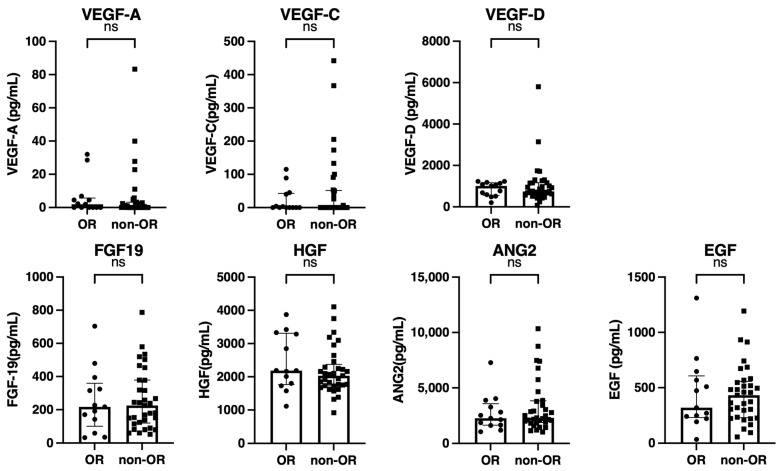
Comparison of the baseline growth factors between patients with and without an objective response (OR) (*n* = 46). Baseline serum VEGF-A, VEGF-C, VEGF-D, FGF-19, HGF, ANG-2, and EGF levels were compared between patients with and without an OR. The bar graph shows the median serum growth factor levels with an interquartile range indicating the error bars. ns, not significant; VEGF, vascular endothelial growth factor; FGF-19, fibroblast growth factor-19; HGF, hepatocyte growth factor; ANG-2, angiopoietin-2; EGF, epidermal growth factor.

As shown in Figure 3, the baseline growth factor levels were also similar between patients with and without an OR.

### 3.3. Changes in Growth Factors between Baseline and Best Overall Response Point in Unresectable HCC Patients with Disease Control by Atezolizumab Plus Bevacizumab

**Figure 4:**
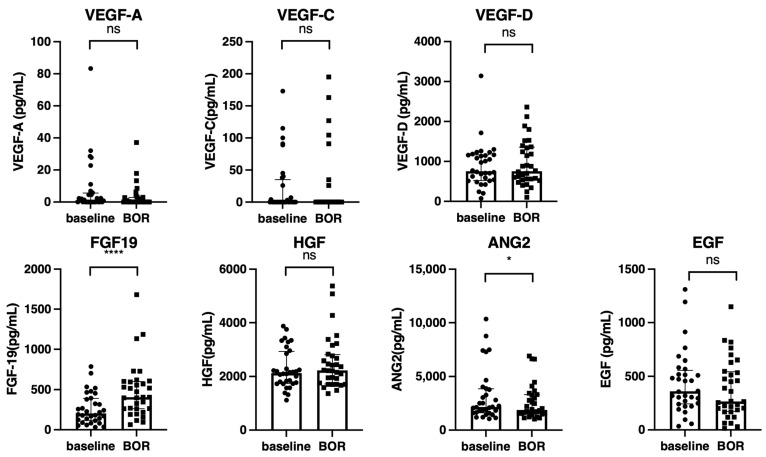
Changes in the growth factors between the baseline and best overall response points (*n* = 32). Serum median VEGF-A, VEGF-C, VEGF-D, FGF-19, HGF, ANG-2, and EGF levels were compared between the baseline and best overall response points in all cohorts. The bar graph shows the median serum growth factor levels with an interquartile range indicating the error bars. Asterisks indicate statistically significant differences (* *p* < 0.05, **** *p* < 0.0001). ns, not significant. VEGF, vascular endothelial growth factor; FGF-19, fibroblast growth factor-19; HGF, hepatocyte growth factor; ANG-2, angiopoietin-2; EGF, epidermal growth factor; BOR, best overall response.

Next, the changes in growth factors between the baseline and best overall response points in 32 patients who achieved CR (*n* = 4), PR (*n* = 9), or SD (*n* = 19) were analyzed. As shown in Figure 4, the FGF-19 level was significantly increased at the best overall response point; in contrast, the ANG2 significantly decreased at the best overall response point.

### 3.4. Changes in Growth Factors between the Best Overall Response Point and PD Point in Unresectable HCC Patients with Disease Control by Atezolizumab Plus Bevacizumab 

**Figure 5:**
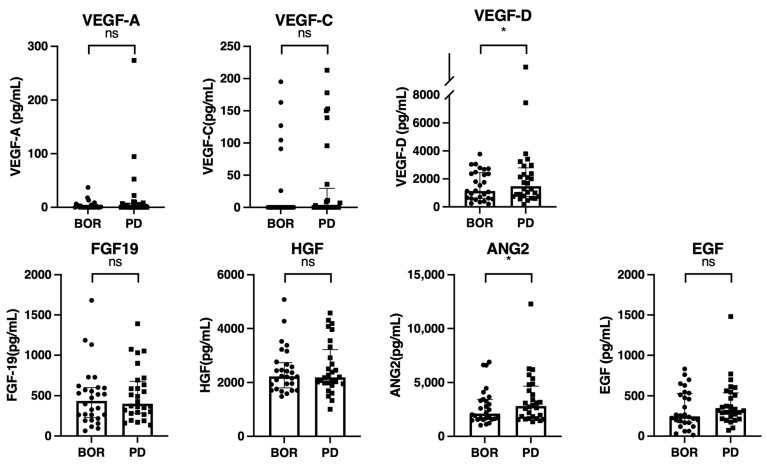
Changes in growth factors between the best overall response points and progressive disease points (*n* = 28). Serum median VEGF-A, VEGF-C, VEGF-D, FGF-19, HGF, ANG-2, and EGF levels were compared between the best overall response and PD points in 28 patients who experienced PD after an initial first response to atezolizumab plus bevacizumab. The bar graph shows the median serum growth factor levels with an interquartile range indicating the error bars. Asterisks indicate statistically significant differences (* *p* < 0.05,). ns, not significant. VEGF, vascular endothelial growth factor; FGF-19, fibroblast growth factor-19; HGF, hepatocyte growth factor; ANG-2, angiopoietin-2; EGF, epidermal growth factor; DC, disease control; PD, progressive disease.

Finally, we analyzed the changes in growth factors between the best overall response point and PD point in 28 patients who achieved disease control with atezolizumab plus bevacizumab; however, they subsequently experienced PD (Table 3, Figure 1). As shown in Figure 5, the VEGF-D and ANG2 levels were significantly increased at the PD point compared to that at the best overall response point.

## 4. Discussion

In this study, we analyzed the changes in serum growth factors before, during, and after atezolizumab plus bevacizumab treatment in patients with unresectable HCC, and found that the baseline growth factor levels were similar between patients with or without disease control, and between patients with or without an OR. An analysis of the patients in the disease control group revealed that serum FGF-19 levels significantly increased at the best overall response point compared to baseline, and serum ANG-2 levels significantly decreased at the best overall response point compared to baseline. Additionally, an analysis of the patients who experienced disease control and subsequent PD revealed that serum VEGF-D and ANG-2 levels significantly increased at the PD point compared with the best overall response point. Thus, there is a possibility that increased serum VEGF-D and ANG-2 levels may contribute to acquiring resistance to atezolizumab plus bevacizumab treatment.

Bevacizumab targets VEGF-A, which predominantly binds VEGFR2, whereas VEGF-C and VEGF-D can also bind to VEGFR2 and cannot be inhibited by bevacizumab [24]. Although there was no statistical difference, serum VEGF-A levels tended to decrease during bevacizumab administration at the best overall response point compared to those at baseline. In this study, serum VEGF-D levels significantly increased at the PD point than at the best overall response point. Thus, the increasing serum VEGF-D level may reactivate VEGFR2-mediated signaling, resulting in the acquisition of resistance to atezolizumab plus bevacizumab. Similar results were reported in colorectal carcinoma patients who received a bevacizumab plus FOLFIRI (folinic acid, 5-fluorouracil, irinotecan) regimen; further, VEGF-A significantly decreased while VEGF-D significantly increased during the treatment process [25]. Importantly, in colorectal cancer patients who failed to respond to first-line therapy with the bevacizumab-containing regimen, the FGFR2 inhibitor ramucirumab, which suppresses not only VEGF-A but also VEGF-C and VEGF-D mediated signaling, showed a better OS in patients with high serum VEGF-D levels than in patients with a low serum VEGF-D level [26]. Taken together, these two colorectal carcinoma studies showed that some patients with colorectal carcinoma developed high VEGF-D levels during VEGF-A inhibitor bevacizumab therapy, and increased VEGF-D might cause resistance to bevacizumab by switching the main source of VEGFR2 activation from VEGF-A to VEGF-D. Therefore, the second-line of ramucirumab combination therapy might be effective in patients with high serum VEGF-D levels, since ramucirumab suppresses both VEGF-A- and VEGF-D-mediated signaling.

The same hypothesis was adopted in the present study. Our results showed that serum VEGF-D levels increased at PD points in patients who experienced PD after a single response to atezolizumab plus bevacizumab treatment. Therefore, second-line therapy using the VEGFR2 inhibitor, ramucirumab, or TKIs, which can suppress VEGFR2-mediated signaling, may be effective for patients with increased VEGF-D. Recently, Shimose et al. reported that the median OR rate in patients with unresectable HCC treated with ramucirumab after atezolizumab plus bevacizumab was significantly higher than that in patients treated with ramucirumab after other therapies (33.3%. vs. 0.0%, *p* = 0.001) [27]. Therefore, we hypothesized that in patients with unresectable HCC, ramucirumab as a second-line therapy after atezolizumab plus bevacizumab might be effective due to the VEGF-D elevation after atezolizumab plus bevacizumab treatment. However, further studies are required to validate this hypothesis.

VEGF-C and VEGF-D are structurally similar and bind to VEGFR 2 and 3 [28]. The reason why VEGF-D, and not VEGF-C, increased at the PD point is unclear, however, there are several hypotheses. VEGF-C and VEGF-D have different binding potentials to VEGFR2 and their levels of expression in tissues are different [29]. Moreover, VEGF-D has a higher angiogenic potential than VEGF-C [30]. Thus, in the case of VEGF-D elevation, HCC patients might be prone to developing resistance to atezolizumab plus bevacizumab. However, further analysis is required to validate this hypothesis. 

In addition, in this study, we observed that ANG-2 significantly increased at the PD point compared to the best overall response point. ANG-2 exerts angiogenesis and tumor growth effects through Tie2-mediated signaling, and is associated with resistance to anti-VEGF therapy [31,32]. In addition, ANG-2 inhibition promotes a proinflammatory tumor microenvironment [31,33]. Thus, increased serum ANG-2 levels could lead to resistance to atezolizumab plus bevacizumab. In this study, ANG-2 significantly decreased at the best overall response point during atezolizumab plus bevacizumab treatment, compared with that at baseline. Thus, further analysis is required to clarify whether the increase in serum ANG-2 levels at the PD point is a cause or effect of tumor growth in unresectable HCC.

The effect of previous systemic therapy on serum growth factors has been not clarified well. Thus, we conducted an additional stratified analysis. As shown in Supplementary Figure S1, serum EGF levels were significantly lower in patients with a history of systemic therapy compared to those without it. In this study, most patients with a history of systemic therapy were treated with lenvatinib. Recently, we reported that median serum EGF levels did not change during and after lenvatinib treatment for unresectable HCC [18]. Thus, these differences might be due to a small number of patients. Thus, further analysis is required. In addition, as shown in Supplementary Figures S2 and S3, baseline growth factors did not affect the objective response and disease control in both groups of patients with or without a history of systemic therapy. Additionally, we conducted additional analysis for changes in growth factors between the best overall response point and the PD points stratified by the history of systemic therapy. As shown in Supplementary Figure S4A, in patients without a history of systemic therapy, VEGF-D increased significantly at the PD point compared to the best overall response point (*p* = 0.0081). Median ANG-2 levels increased marginally at the PD point compared to the best overall response point (*p* = 0.0681). As shown in Supplementary Figure S4B, in patients with a history of systemic therapy, VEGF-D and ANG-2 increased at the PD point compared to the best overall response point; however, it was not significant. Thus, the association between increased VEGF-D and ANG-2 levels and the acquisition of resistance to atezolizumab and bevacizumab for unresectable HCC is more relevant in patients without a history of systemic therapy. However, the included patients were relatively limited, therefore, further comparison studies with a large number of patients are required.

In addition, whether VEGF-D and ANG2 are associated with an initial resistance to atezolizumab and bevacizumab treatment for unresectable HCC has been not clarified well. Thus, we compared the ANG-2 and VEGF-D levels between the baseline and PD points in patients with the best overall response of PD. As shown in Supplementary Figure S5, ANG2 and VEGF-D levels were similar between the baseline and PD points in patients with the best overall response of PD. In addition, as shown in Figure 2, the baseline growth factor levels were similar between patients with or without the best overall response of PD. Thus, factors other than ANG-2 and VEGF-D might contribute to the initial resistance to atezolizumab and bevacizumab. Thus, further analysis is required for clarification.

In this study, a total of 34.8% experienced an interruption of bevacizumab. Interruption by the anti-VEGF-A antibody of bevacizumab might affect the changes in growth factors; thus, we analyzed the effect of bevacizumab interruption on changes in growth factors between the best overall response points and the PD points. As shown in Supplementary Figure S6, in patients without bevacizumab interruption, VEGF levels were significantly or marginally significantly increased at the PD point compared to those at the best overall response point [(VEGF-A (*p* = 0.0312), VEGF-C (*p* = 0.0156), and VEGF-D (*p* = 0.0637)]. Thus, in patients with continuous administration of an anti-VEGF-A antibody of bevacizumab, increased VEGFs, including VEGF-A, C, and D, might contribute to acquiring resistance to atezolizumab and bevacizumab more prominently than those with bevacizumab interruption. However, because the included number of patients was limited, a study with a large sample size is required to validate this result.

This study had several limitations. This was a retrospective single-center study. The number of patients included in the study was relatively small. Especially, the number of patients who experienced PD after the first response to atezolizumab plus bevacizumab was limited. Thus, a multicenter prospective study is required, shortly, to validate these results.

## 5. Conclusions

Serum VEGF-D and ANG-2 levels were significantly increased at the PD point compared to the best overall response point. Therefore, increased serum VEGF-D and ANG-2 levels might contribute to resistance to atezolizumab plus bevacizumab for unresectable HCC, and might be remarkable target molecules in subsequent salvage therapies.

## Figures and Tables

**Table 1:** Baseline patient characteristics in patients with and without DC.

	Included Patients	DC (+) *n* = 32	DC (−) *n* = 14	*p*-Value
	*n* = 46			
Age (years), median (range)	72 (31–84)	68 (47–74)	75 (47–81)	0.0237
Sex (male/female)	37/9	27/5	10/4	0.4226
Etiology, *n* (%)				0.5596
HBV	15 (32.6%)	12 (37.5%)	3 (21.4%)	
HCV	6 (13.0%)	4 (12.5%)	2 (14.3%)	
NBNC	25 (54.3%)	16 (50.0%)	9 (64.3%)	
Liver condition				0.7539
LC	22 (47.8%)	16 (50%)	6 (42.9%)	
CH	24 (52.2%)	16 (50%)	8 (57.1%)	
BCLC stage, *n* (%)				>0.9999
B	16 (34.8%)	11 (34.4%)	5 (35.7%)	
C	30 (65.2%)	21 (65.6%)	9 (64.3%)	
Child–Pugh class, *n* (%)				>0.9999
A	46 (100%)	32 (100%)	14 (100%)	
B	0 (0%)	0 (0%)	0 (0%)	
Child–Pugh score, *n* (%)				0.3161
5	30 (65.2%)	19 (59.4%)	11 (78.6%)	
6	16 (34.8%)	13 (40.6%)	3 (21.4%)	
Biochemical analysis				
Platelets, ×10^4^/μL	15.3 (6.1–54)	14.45 (6.1–46.4)	16.45 (8.5–54)	0.5351
ALT, IU/L	24.5 (7–278)	19 (7–278)	42.5 (19–122)	0.0010
Cr, mg/dL	0.825 (0.097–2.28)	0.88 (0.52–2.28)	0.705 (0.097–1.44)	0.0913
AFP, ng/mL	24.05 (2.3–57,125.2)	19.35 (2.3–57,125.2)	221.6 (2.4–15,009.5)	0.2023
PIVKA-II, mAU/mL	886.5 (19–213,066)	1006 (19–213,066)	881.5 (25–110,159)	0.8926
History of operation	24 (52.2%)	14 (43.8%)	10 (71.4%)	0.1141
History of RFA	14 (30.4%)	9 (28.1%)	5 (35.7%)	0.7308
History of TACE	25 (54.3%)	18 (56.3%)	7 (50%)	0.7553
History of systemic therapy	24 (52.2%)	18 (56.3%)	6 (42.9%)	0.5252
No treatment history	8 (17.4%)	6 (18.8%)	2 (14.3%)	>0.9999
Interruption of bevacizumab	16 (34.8%)	13 (40.6%)	3 (21.4%)	0.3161

HBV, hepatitis B virus infection; HCV, hepatitis C virus infection; NCBC, non-hepatitis B non-hepatitis C infection; BCLC, Barcelona Clinic Liver Cancer; ALT, alanine aminotransferase; Cr, creatinine; AFP, α-fetoprotein; PIVKA-II, protein induced by vitamin K absence-II; DC, disease control.

**Table 2:** Baseline characteristics of patients with or without an OR.

	Included Patients	OR (+) *n* = 13	OR (−) *n* = 33	*p*-Value
	*n* = 46			
Age (years), median (range)	72 (31–84)	68 (47–74)	70 (31–84)	0.0236
Sex (male/female)	37/9	11/2	26/7	>0.9999
Etiology, *n* (%)				0.2558
HBV	15 (32.6%)	5 (38.5%)	10 (30.3%)	
HCV	6 (13.0%)	0 (0%)	6 (18.2%)	
NBNC	25 (54.3%)	8 (61.5%)	17 (51.5%)	
Liver condition				0.7462
LC	22 (47.8%)	7 (53.8%)	15 (45.5%)	
CH	24 (52.2%)	6 (46.2%)	18 (54.5%)	
BCLC stage, *n* (%)				0.7441
B	16 (34.8%)	5 (38.5%)	11 (33.3%)	
C	30 (65.2%)	8 (61.5%)	22 (66.7%)	
Child–Pugh class, *n* (%)				>0.9999
A	46 (100%)	13 (100%)	33 (100%)	
B	0 (0%)	0 (0%)	0 (0%)	
Child–Pugh score, *n* (%)				0.3095
5	30 (65.2%)	7 (53.8%)	23 (69.7%)	
6	16 (34.8%)	6 (46.2%)	10 (30.3%)	
Biochemical analysis				
latelets, ×10^4^/μL	15.3 (6.1–54)	17.2 (7.6–46.4)	15.2 (6.1–54)	0.9568
ALT, IU/L	24.5 (7–278)	19 (7–43)	31 (7–278)	0.0639
Cr, mg/dL	0.825 (0.097–2.28)	0.78 (0.52–1.42)	0.89 (0.097–2.28)	0.3487
AFP, ng/mL	24.05 (2.3–57,125.2)	18.1 (2.3–2945.4)	80.1 (2.3–57,125.2)	0.4145
PIVKA-II, mAU/mL	886.5 (19–213,066)	277 (19–3217)	1616 (20–213,066)	0.0699
History of operation	24 (52.2%)	6 (46.2%)	18 (54.5%)	0.7462
History of RFA	14 (30.4%)	5 (38.5%)	9 (27.3%)	0.4934
History of TACE	25 (54.3%)	8 (61.5%)	17 (51.5%)	0.7437
History of systemic therapy	24 (52.2%)	9 (69.2%)	15 (45.5%)	0.1968
No treatment history	8 (17.4%)	3 (23.1%)	5 (15.2%)	0.6689
Interruption of bevacizumab	16 (34.8%)	4 (30.8%)	12 (36.4%)	>0.9999

HBV, hepatitis B virus infection; HCV, hepatitis C virus infection; NCBC, non-hepatitis B non-hepatitis C infection; BCLC, Barcelona Clinic Liver Cancer; ALT, alanine aminotransferase; Cr, creatinine; AFP, α-fetoprotein; PIVKA-II; protein induced by vitamin K absence-II; OR, objective response.

**Table 3:** Baseline characteristics of patients who experienced PD after CR, PR, or SD with atezolizumab plus bevacizumab.

	Included Patients
	*n* = 28
Age (years), median (range)	70 (31–84)
Sex (male/female)	23/5
Treatment response	
CR	2 (7.1%)
PR	7 (25.0%)
SD	19 (67.9%)
Etiology, *n* (%)	
HBV	9 (32.1%)
HCV	4 (14.3%)
NBNC	15 (53.6%)
Liver condition	
LC	14 (50.0%)
CH	14 (50.0%)
BCLC stage, *n* (%)	
B	10 (35.7%)
C	18 (64.3%)
Child–Pugh class, *n* (%)	
A	28 (100%)
B	0 (0%)
Child–Pugh score, *n* (%)	
5	16 (57.1%)
6	12 (42.9%)
Biochemical analysis	
Platelets, ×10^4^/μL	13.7 (6.1–41.3)
ALT, IU/L	23 (7–278)
Cr, mg/dL	0.89 (0.52–2.28)
AFP, ng/mL	19.4 (2.3–57,125.2)
PIVKA-II, mAU/mL	1852 (20–213,066)
History of operation	11 (39.3%)
History of RFA	8 (28.6%)
History of TACE	15 (53.6%)
History of systemic therapy	15 (53.6%)
No treatment history	4 (14.3%)
Interruption of bevacizumab	13 (46.4%)

CR, complete response; PR, partial response; SD, stable disease; HBV, hepatitis B virus infection; HCV, hepatitis C virus infection; NCBC, non-hepatitis B non-hepatitis C infection; BCLC, Barcelona Clinic Liver Cancer; ALT, alanine aminotransferase; Cr, creatinine; AFP, α-fetoprotein; PIVKA-II; protein induced by vitamin K absence-II; DC, disease control; PD, progressive disease.

## Data Availability

All data generated or analyzed during this study are included in this article. Further inquiries can be directed to the corresponding author.

## Supplementary Materials

The following supporting information can be downloaded at: https://www.mdpi.com/article/10.3390/cancers15030593/s1, Figure S1: Comparison of baseline growth factors between patients with or without a history of systemic therapy; Figure S2: (A) Comparison of baseline growth factors between patients with and without an objective response in patients without a history of systemic therapy (*n* = 22); (B) Comparison of baseline growth factors between patients with and without an objective response in patients with a history of systemic therapy (*n* = 24); Figure S3: (A) Comparison of baseline growth factors between patients with or without disease control, without a history of systemic therapy (*n* = 22); (B) Comparison of baseline growth factors between patients with or without disease control in patients with a history of systemic therapy (*n* = 24); Figure S4: (A) Changes in growth factors between the best overall response points and progressive disease in patients without a history of systemic therapy (*n* = 13); (B) Changes in growth factors between the best overall response points and progressive disease in patients with a history of systemic therapy (*n* = 15); Figure S5: Changes in serum ANG2 and VEGF-D levels between baseline and the progressive disease points in patients with the best overall response of progressive disease (*n* = 14); Figure S6: (A) Changes in growth factors between the best overall response points and progressive disease in patients who experienced bevacizumab interruption (*n* = 13); (B) Changes in growth factors between the best overall response points and progressive disease in patients who did not experience bevacizumab interruption (*n* = 15).
