# Supplementary material for: Changes in Serum Growth Factors during Resistance to Atezolizumab Plus Bevacizumab Treatment in Patients with Unresectable Hepatocellular Carcinoma
Source: Cancers (Basel). 2023 Jan 18;15(3):593. doi: 10.3390/cancers15030593 (PMC9913372; doi:10.3390/cancers15030593)
Supplement: Supplementary file 1 [file cancers-15-00593-s001.zip › cancers-2125132-supplementary.pdf]

Figure S1 Comparison of baseline growth factors between patients with or without a history of systemic therapy

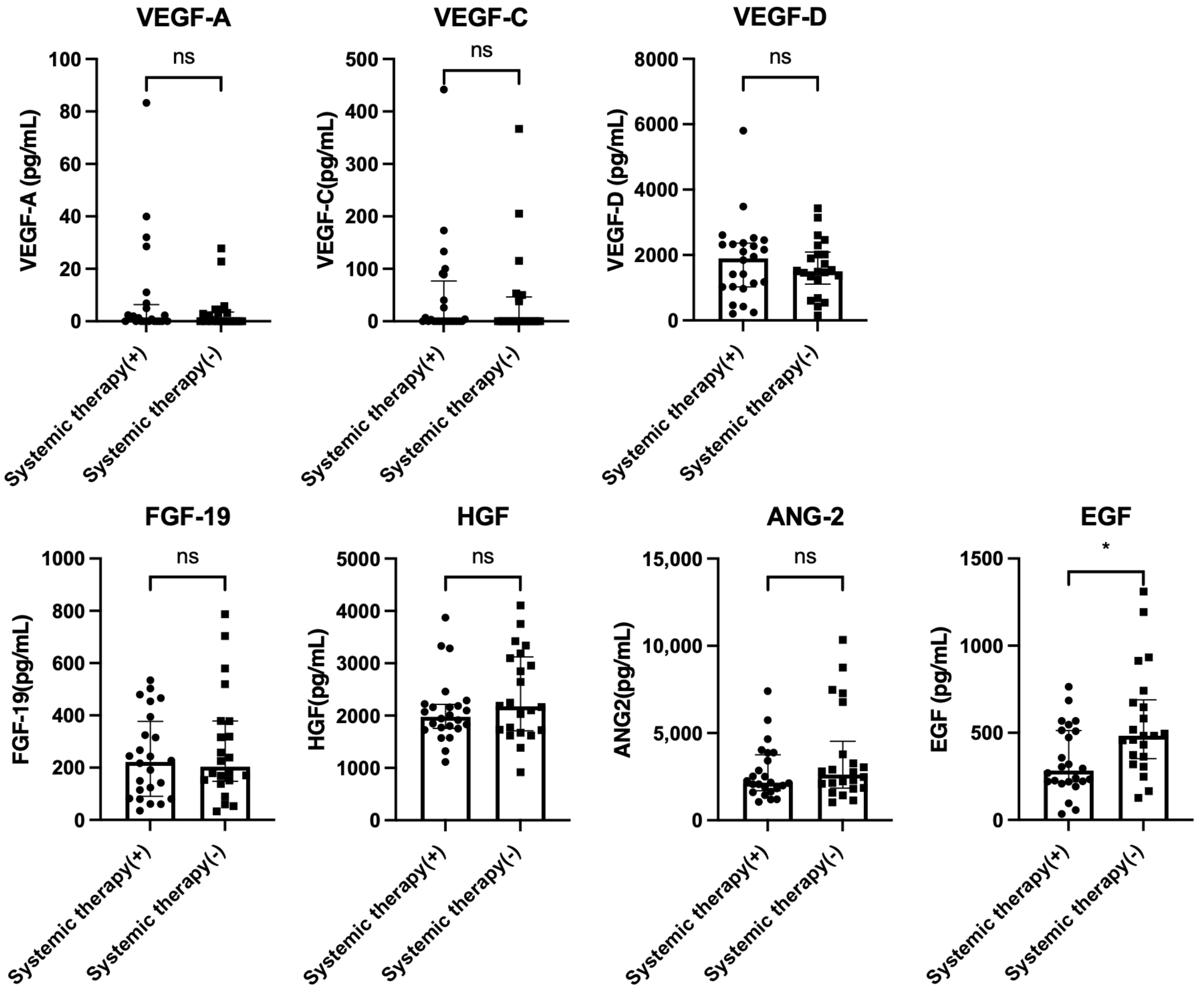

Baseline serum VEGF-A, VEGF-C, VEGF-D, FGF-19, HGF, ANG-2, and EGF levels were compared between patients with or without a history of systemic therapy. The bar graph shows median serum growth factor levels with an interquartile range indicating the error bars. Asterisks indicate statistically significant differences (* $p < 0.05$). Ns, not significant; VEGF, vascular endothelial growth factor; FGF-19, fibroblast growth factor-19; HGF, hepatocyte growth factor; ANG-2, an-giopoietin-2; EGF, epidermal growth factor

Figure S2 A Comparison of baseline growth factors between patients with and without objective response in patients without a history of systemic therapy (n=22)

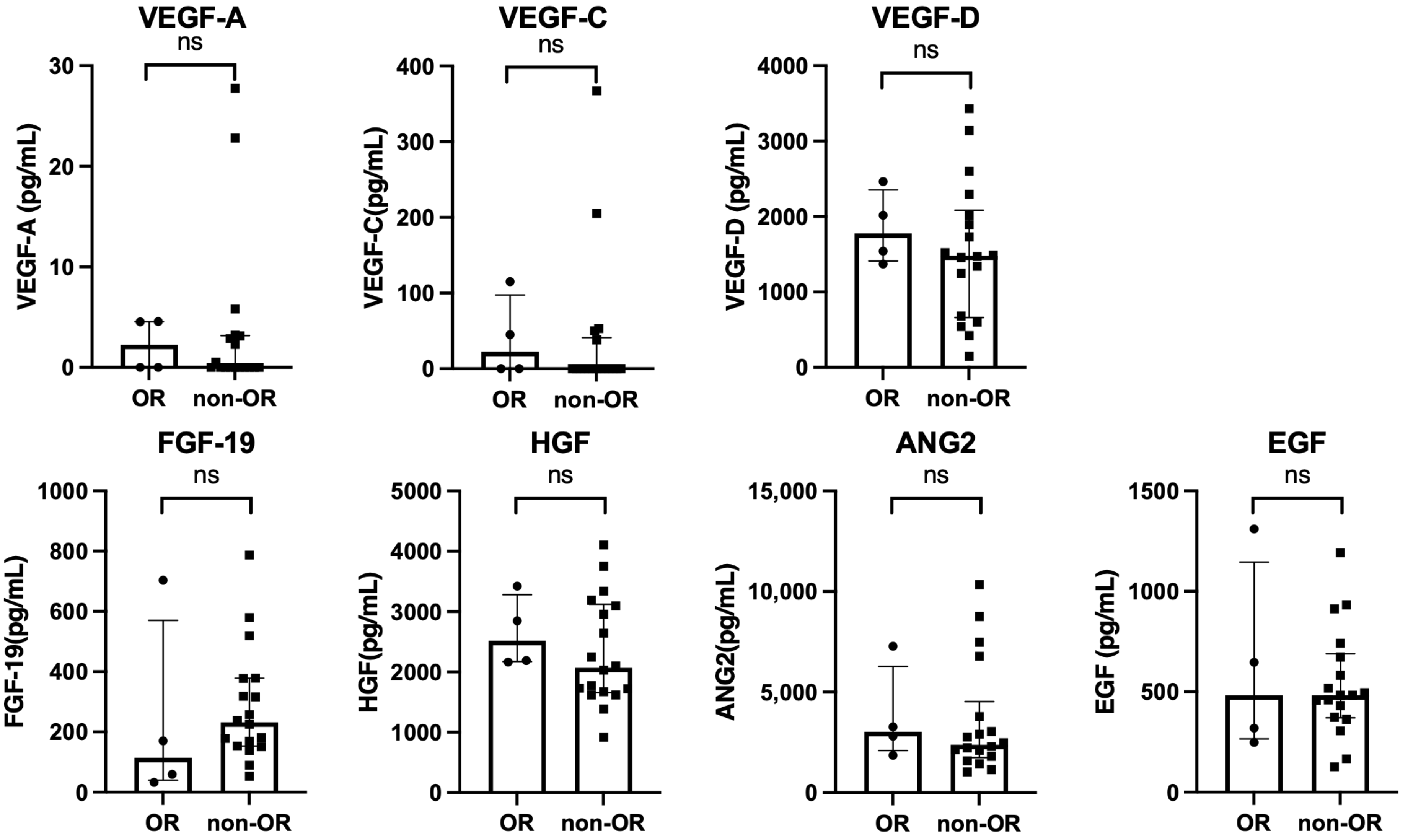

Baseline serum VEGF-A, VEGF-C, VEGF-D, FGF-19, HGF, ANG-2, and EGF levels were compared between patients with or without an OR in patients without a history of systemic therapy. The bar graph shows median serum growth factor levels with an interquartile range indicating the error bars. Ns, not significant; VEGF, vascular endothelial growth factor; FGF-19, fibroblast growth factor-19; HGF, hepatocyte growth factor; ANG-2, angiopoietin-2; EGF, epidermal growth factor

Figure S2 B Comparison of baseline growth factors between patients with and without objective response in patients with a history of systemic therapy (n=24)

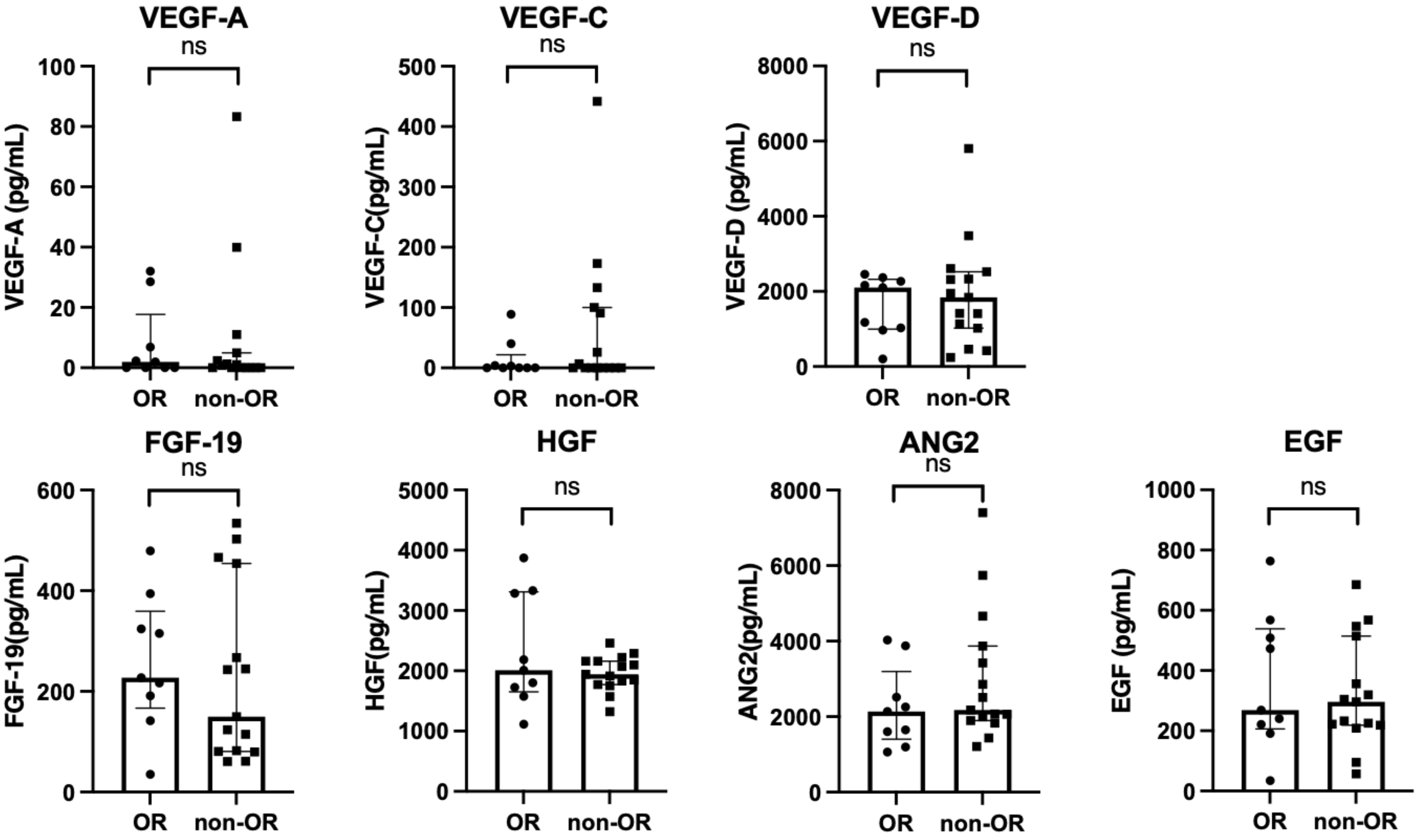

Baseline serum VEGF-A, VEGF-C, VEGF-D, FGF-19, HGF, ANG-2, and EGF levels were compared between patients with or without an OR in patients with a history of systemic therapy. The bar graph shows median serum growth factor levels with an interquartile range indicating the error bars. Ns, not significant; VEGF, vascular endothelial growth factor; FGF-19, fibroblast growth factor-19; HGF, hepatocyte growth factor; ANG-2, angiopoietin-2; EGF, epidermal growth factor

Figure S3A Comparison of baseline growth factors between patients with or without disease control without a history of systemic therapy (n=22)

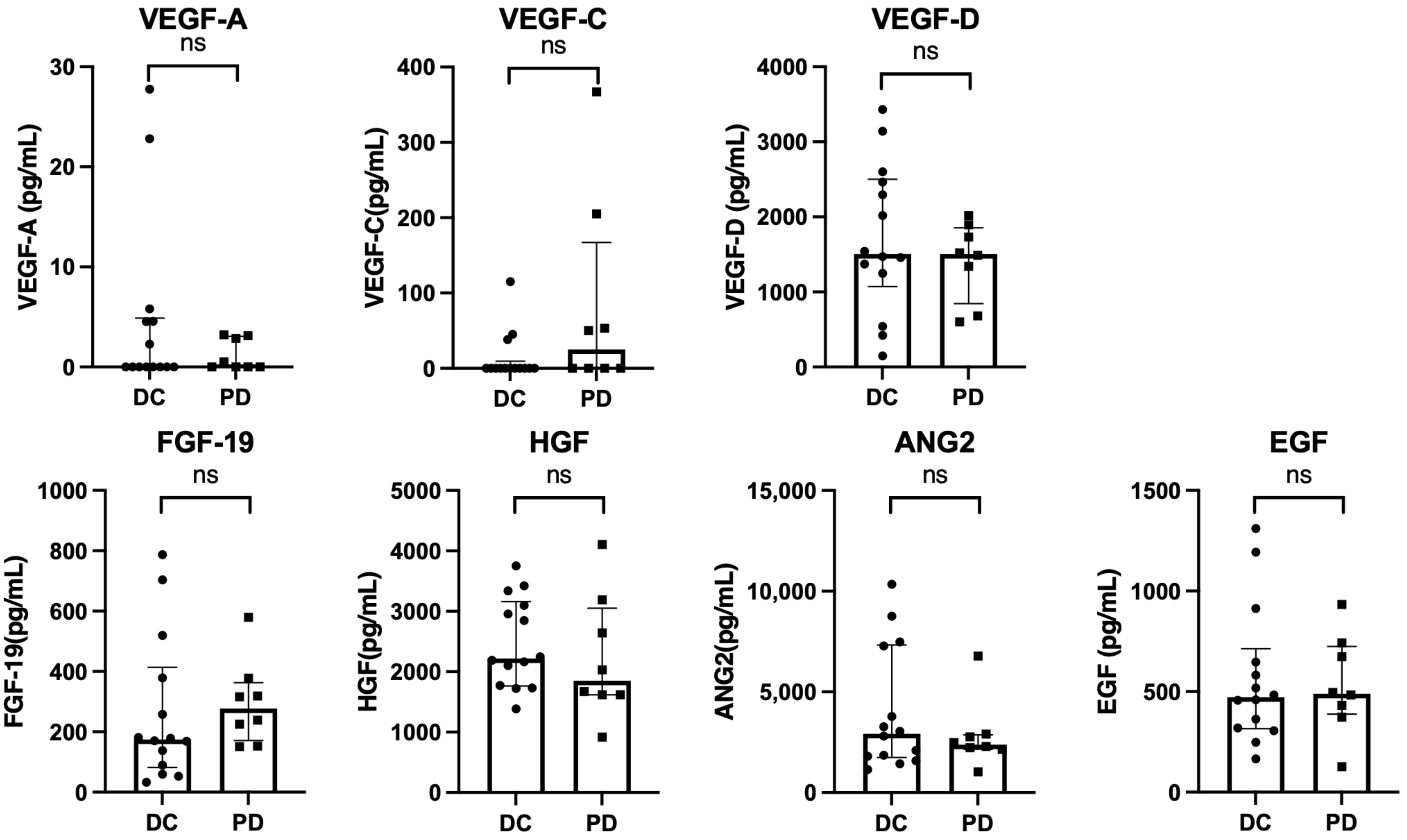

Baseline serum VEGF-A, VEGF-C, VEGF-D, FGF-19, HGF, ANG2, and EGF levels were compared between patients with or without disease control in patients without history a systemic therapy. The bar graph shows median serum growth factor levels with an interquartile range indicating the error bars. Ns, not significant; VEGF, vascular endothelial growth factor; FGF-19, fibroblast growth factor-19; HGF, hepatocyte growth factor; ANG-2, angiopoietin-2; EGF, epidermal growth factor; DC, disease control; PD, progressive disease

Figure S3 B Comparison of baseline growth factors between patients with or without disease control in patients with a history of systemic therapy (n=24)

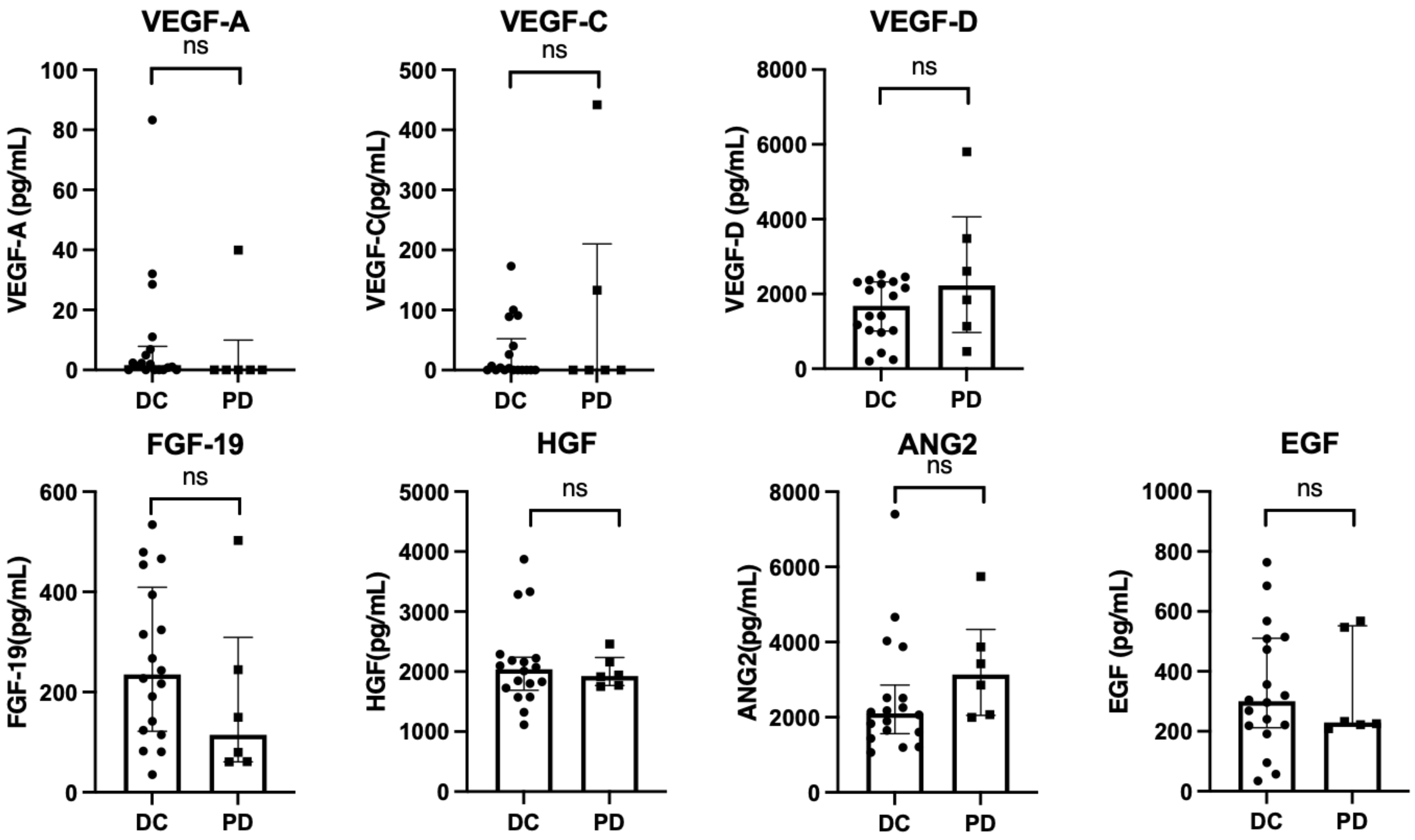

Baseline serum VEGF-A, VEGF-C, VEGF-D, FGF-19, HGF, ANG2, and EGF levels were compared between patients with or without disease control in patients with history of a systemic therapy. The bar graph shows median serum growth factor levels with an interquartile range indicating the error bars. Asterisks indicate statistically significant differences.VEGF, vascular endothelial growth factor; FGF-19, fibroblast growth factor-19; HGF, hepatocyte growth factor; ANG-2, angiopoietin-2; EGF, epidermal growth factor; DC, disease control; PD, progressive disease

Figure S4 A Changes in growth factors between best overall response points and progressive disease in patients without a history of systemic therapy (n=13)

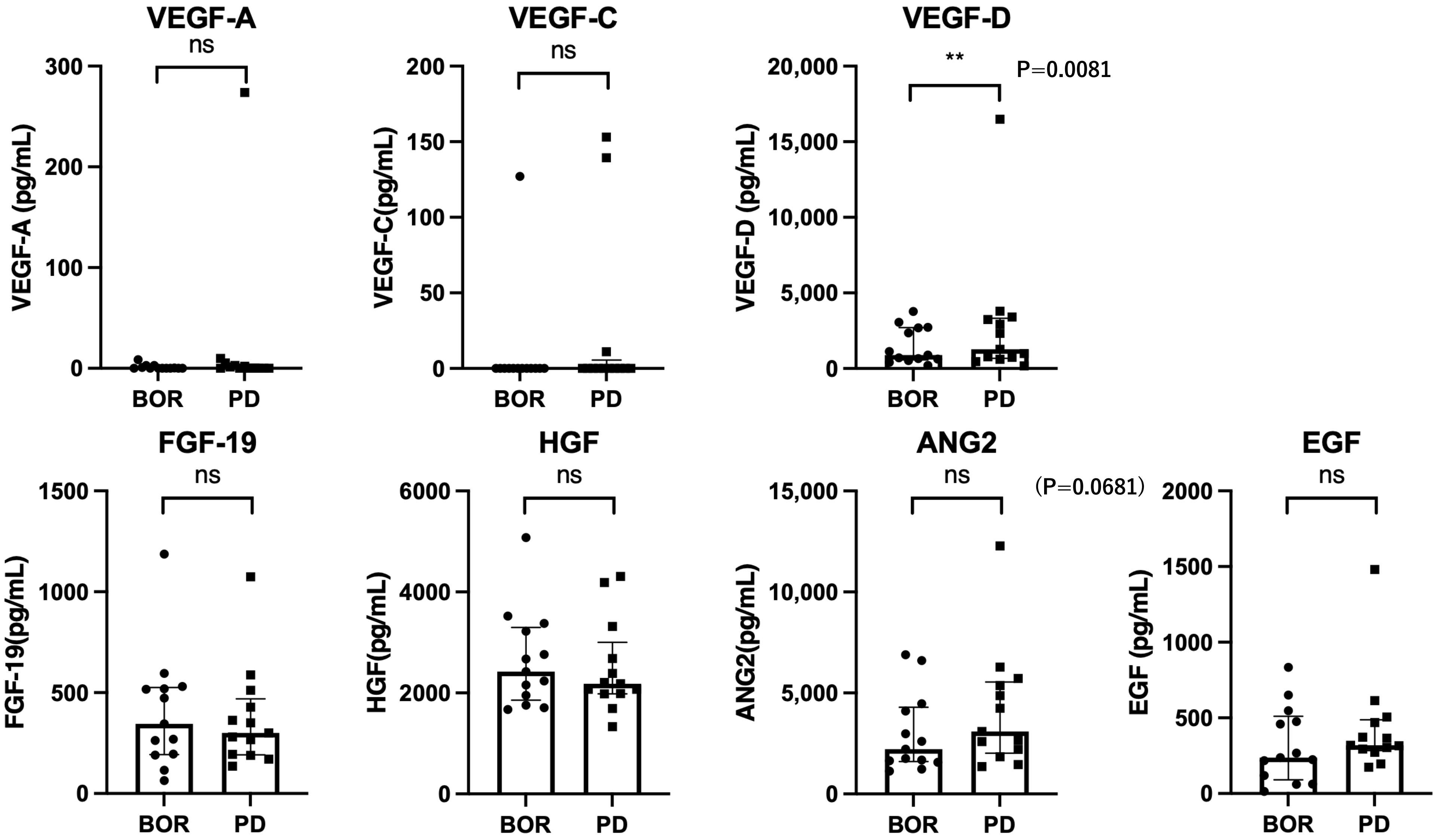

Serum median VEGF-A, VEGF-C, VEGF-D, FGF-19, HGF, ANG2, and EGF levels were compared between best of response and PD points in patients without a history of systemic therapy. The bar graph shows median serum growth factor levels with an interquartile range indicating the error bars. Asterisks indicate statistically significant differences ** p < 0.01). Ns, not significant; VEGF, vascular endothelial growth factor; FGF-19, fibroblast growth factor-19; HGF, hepatocyte growth factor; ANG-2, angiopoietin-2; EGF, epidermal growth factor; BOR, best overall response; PD, progressive disease

Figure S4 B Changes in growth factors between best overall response points and progressive disease in patients with a history of systemic therapy (n=15)

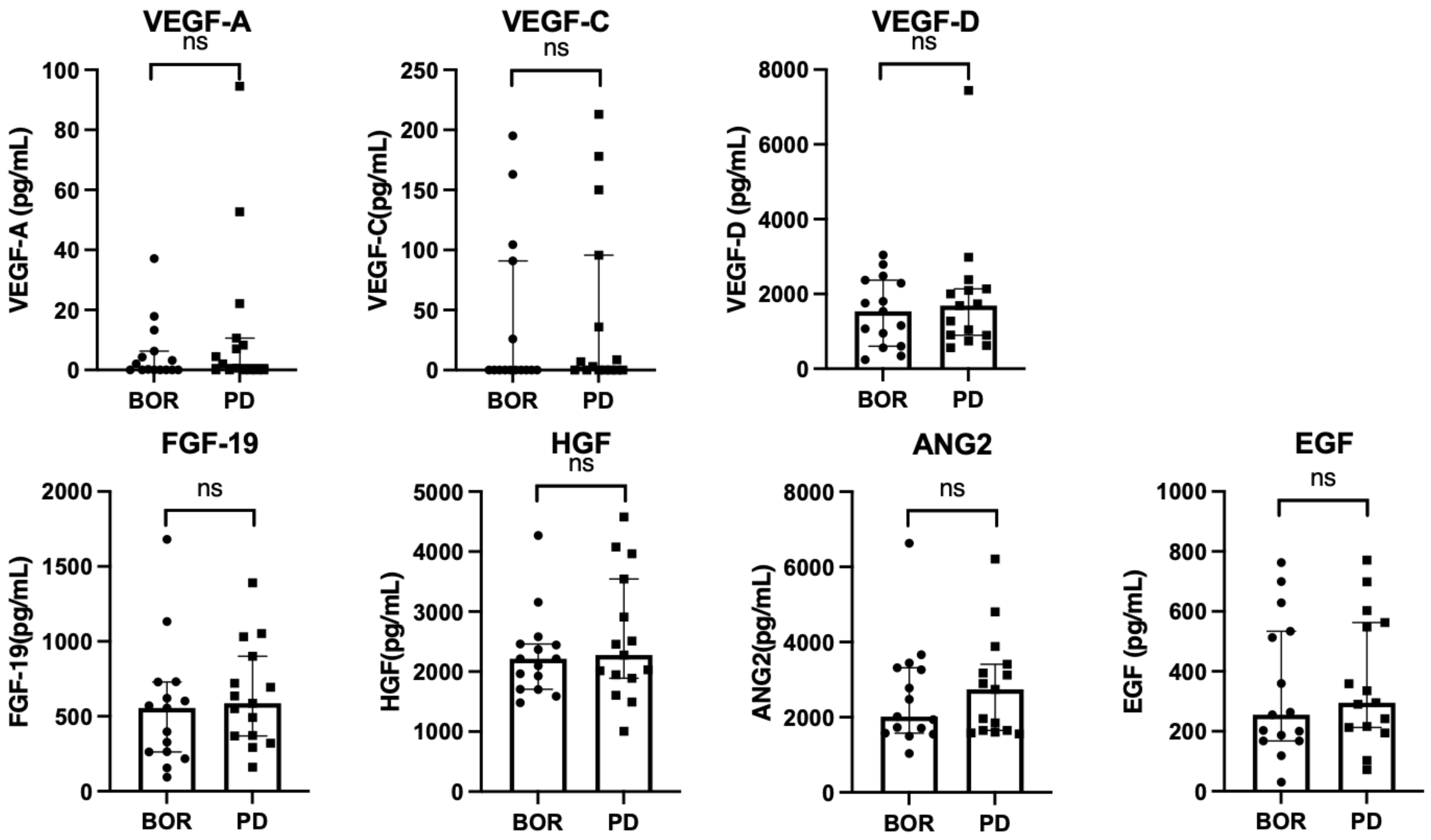

Serum median VEGF-A, VEGF-C, VEGF-D, FGF-19, HGF, ANG2, and EGF levels were compared between the best response and PD points in patients with a history of systemic therapy. The bar graph shows median serum growth factor levels with an interquartile range indicating the error bars. Ns, not significant; VEGF, vascular endothelial growth factor; FGF-19, fibroblast growth factor-19; HGF, hepatocyte growth factor; ANG-2, angiopoietin-2; EGF, epidermal growth factor; BOR, best overall response; PD, progressive disease

Figure S5 Changes in serum ANG2 and VEGF-D levels between baseline and progressive disease points in patients with the best overall response of progressive disease (n=14)

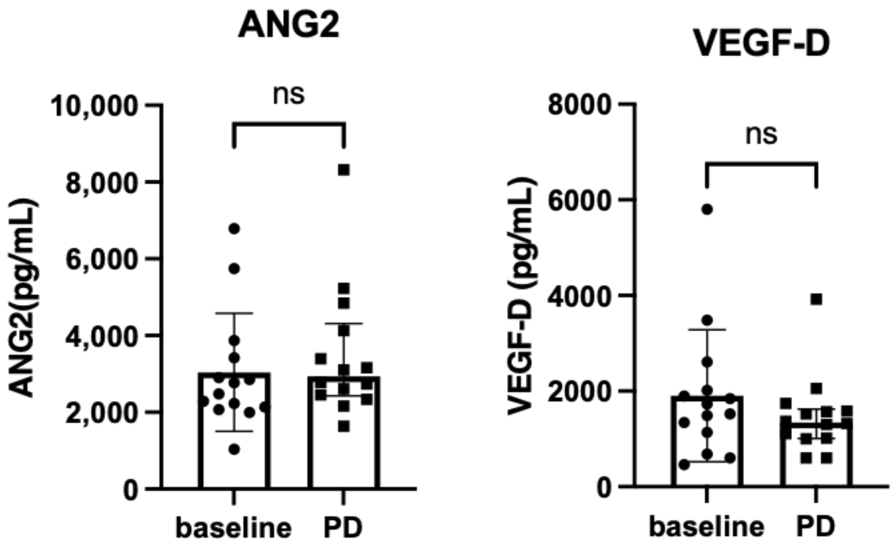

Serum median VEGF-A and VEGF-D levels were compared between baseline and progressive disease points in patients with the best response at progressive disease. The bar graph shows median serum growth factor levels with an interquartile range indicating the error bars. Ns, not significant; VEGF, vascular endothelial growth factor; FGF-19, fibroblast growth factor-19; HGF, hepatocyte growth factor; ANG-2, an-giopoietin-2; EGF, epidermal growth factor; PD, progressive disease

Figure S6A Changes in growth factors between best overall response points and progressive disease in patients who experienced bevacizumab interruption (n=13)

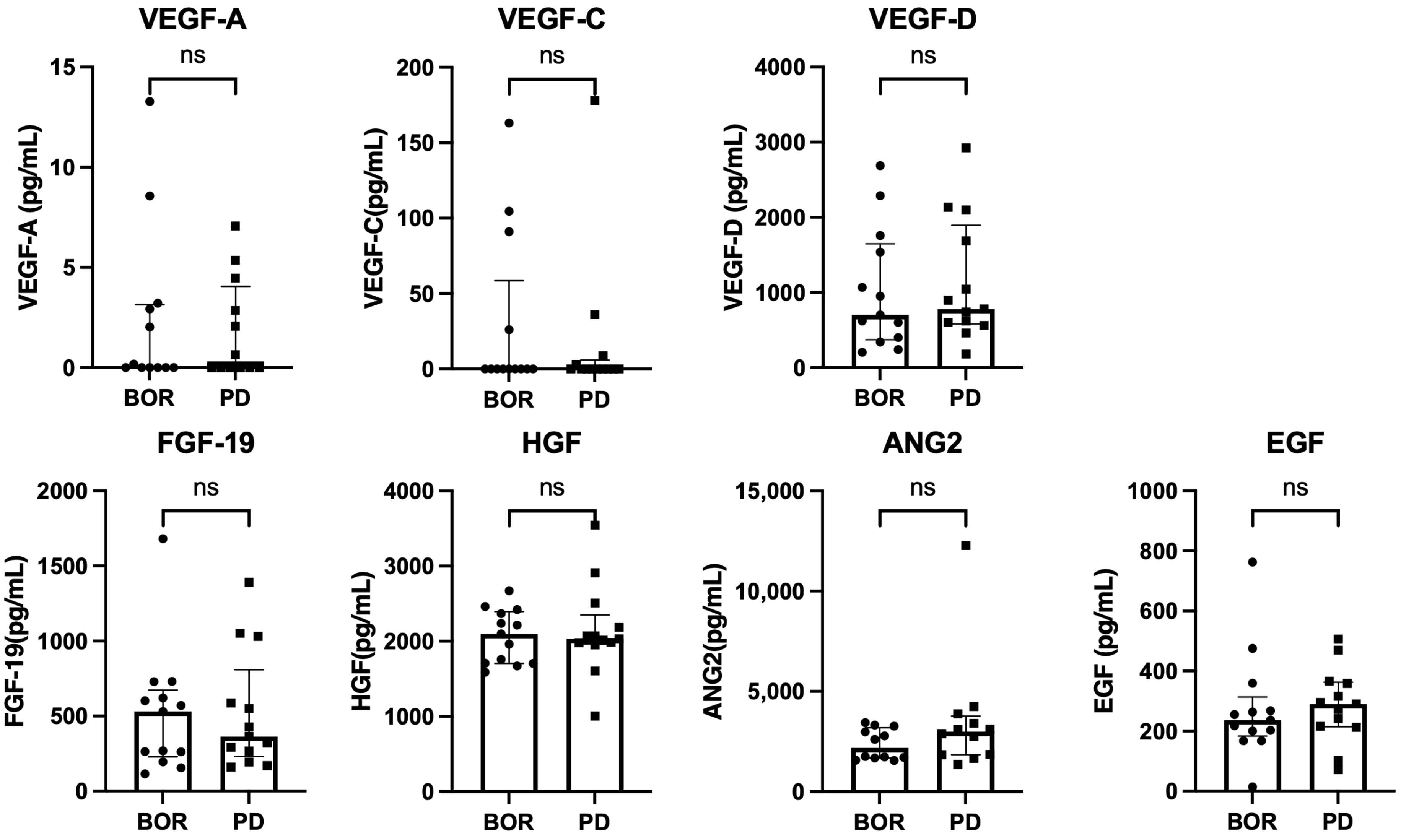

Serum median VEGF-A, VEGF-C, VEGF-D, FGF-19, HGF, ANG-2, and EGF levels were compared between best of response and PD points in patients who experienced bevacizumab interruption. The bar graph shows median serum growth factor levels with an interquartile range indicating the error bars. Ns, not significant; VEGF, vascular endothelial growth factor; FGF-19, fibroblast growth factor-19; HGF, hepatocyte growth factor; ANG-2, angiopoietin-2; EGF, epidermal growth factor; BOR, best overall response; PD, progressive disease

Figure S6B Changes in growth factors between best overall response points and progressive disease in patients who did not experience bevacizumab interruption (n=15)

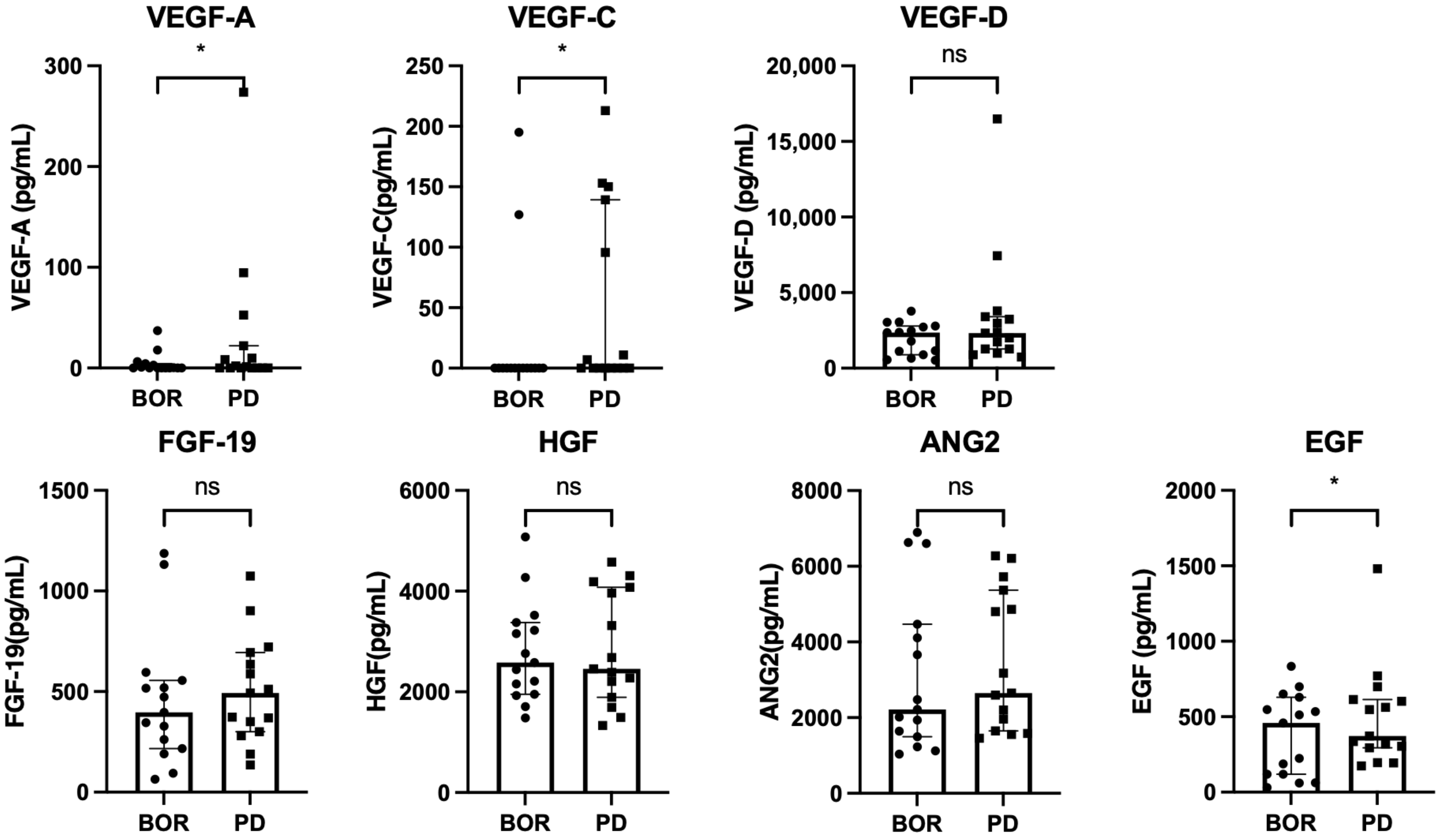

Serum median VEGF-A, VEGF-C, VEGF-D, FGF-19, HGF, ANG-2, and EGF levels were compared between best response and PD points in patients who did not experience bevacizumab interruption. The bar graph shows median serum growth factor levels with an interquartile range indicating the error bars. Asterisks indicate statistically significant differences (* $p < 0.05$). Ns, not significant; VEGF, vascular endothelial growth factor; FGF-19, fibroblast growth factor-19; HGF, hepatocyte growth factor; ANG-2, angiopoietin-2; EGF, epidermal growth factor; BOR, best overall response; PD, progressive disease
